# Comparison of Buyang Huanwu granules and Naoxintong capsules in the treatment of stable angina pectoris: rationale and design of a randomized, blinded, multicentre clinical trial

**DOI:** 10.1186/s13063-021-05914-1

**Published:** 2022-01-21

**Authors:** Yu Wang, Yuhan Xu, Ling Zhang, Shuwei Huang, Liping Dou, Jiehong Yang, Wei Fu, Peng Zhou, Haitong Wan

**Affiliations:** 1 Department of Gastroenterology, Affiliated Hangzhou First People’s Hospital, Zhejiang University School of Medicine, Hangzhou, China; 2 Institute of Cardio-cerebrovascular Disease, Zhejiang Chinese Medical University, Hangzhou, China; 3 Department of Cardiology, First Affiliated Hospital of Zhejiang Chinese Medical University, Hangzhou, China; 4 Department of Cardiology, Second Affiliated Hospital of Zhejiang Chinese Medical University, Hangzhou, China; 5 School of Basic Medical Sciences and Public Health, Zhejiang Chinese Medical University, Hangzhou, China; 6 Department of Cardiac-Cerebral Diseases, Yinchuan Cardiac-Cerebral Treatment Internet Hospital, Yinchuan, China; 7 Institute of Brain and Heart CO Treatment, Zhejiang Chinese Medical University, Hangzhou, China

**Keywords:** Stable angina pectoris, Traditional Chinese medicine, Buyang Huanwu granules, Naoxintong capsules, Randomized controlled trial

## Abstract

**Background:**

Stable angina pectoris (SAP) currently seriously threatens the health of humans, and mortality is continuously rising. Current treatment strategies mainly include pharmaceutical therapy and revascularization. In China, Buyang Huanwu granules (BYHW) and Naoxintong capsules (NXT) have been used in the treatment of SAP, but it is not clear which agent is better in terms of relieving symptoms and improving quality of life. Therefore, we designed a clinical trial to compare the efficacy and safety of NXT and BYHW in the treatment of SAP.

**Methods:**

This is a randomized, blinded, parallel controlled, multicentre clinical trial protocol. On the basis of standardized Western medicine treatment, a total of 128 SAP patients will be randomly divided into intervention group 1 (NXT group), intervention group 2 (BYHW group), and a control group (placebo group) at a 2:1:1 ratio. A 2-week run-in period is required prior to randomization, and a 1-week baseline period and 4-week treatment period are included in this study. The primary outcome is the efficacy rate of stable angina symptom score improvement; the secondary outcomes include the effect on electrocardiograms, Seattle Angina Questionnaire scores, and nitroglycerine consumption.

**Discussion:**

This study will evaluate the efficacy and safety of NXT and BYHW in the treatment of SAP. The results will provide critical evidence for using Chinese herbal medicines to treat SAP.

**Trial registration:**

Chinese Clinical Trials Registry ChiCTR1800015191. Registered on 13 March 2018. http://www.chictr.org.cn/showproj.aspx?proj=25818. All the registration items can be found within the protocol.

## Administrative information

Note: the numbers in curly brackets in this protocol refer to SPIRIT checklist item numbers. The order of the items has been modified to group similar items (see http://www.equator-network.org/reporting-guidelines/spirit-2727-statement-defining-standard-protocol-items-for-clinical-trials/).

**Table:** 

Title {1}	Protocol for a randomized, blinded, multicenter clinical trial to compare efficacy rate of stable angina symptom score improvement between Buyang Huanwu granules and Naoxintong capsules for adults with stable angina pectoris
**Trial registration {2a and 2b}.**	Chinese Clinical Trials Registry, ChiCTR1800015191, registered on 13 March 2018. http://www.chictr.org.cn/showproj.aspx?proj=25818 All the registration items can be found within the protocol.
**Protocol version {3}**	December 22, 2017; version 2.0.
**Funding {4}**	This study is supported by the National Natural Science Foundation of China (No.81630105), the National Key R&D Program for Modernization of Traditional Chinese Medicine (2019YFC1708600, 2019YFC17086003), and Zhejiang Province High-level Talents Project (No. 2019R51002).
**Author details {5a}**	Yu Wang (shared first authorship) Department of Gastroenterology, Affiliated Hangzhou First People's Hospital, Zhejiang University School of Medicine, Hangzhou, China Institute of Cardio-cerebrovascular Disease, Zhejiang Chinese Medical University, Hangzhou, China Yuhan Xu (shared first authorship) Institute of Cardio-cerebrovascular Disease, Zhejiang Chinese Medical University, Hangzhou, China Ling Zhang Institute of Cardio-cerebrovascular Disease, Zhejiang Chinese Medical University, Hangzhou, China Shuwei Huang Department of Cardiology, First Affiliated Hospital of Zhejiang Chinese Medical University, Hangzhou, China Liping Dou Department of Cardiology, Second Affiliated Hospital of Zhejiang Chinese Medical University, Hangzhou, China Jiehong Yang School of Basic Medical Sciences and Public Health, Zhejiang Chinese Medical University, Hangzhou, China Wei Fu Department of Cardiac-Cerebral Diseases, Yinchuan Cardiac-Cerebral Treatment Internet Hospital, Yinchuan, China Peng Zhou Institute of Brain and Heart CO Treatment, Zhejiang Chinese Medical University, Hangzhou, China Haitong Wan (corresponding author) Institute of Cardio-cerebrovascular Disease, Zhejiang Chinese Medical University, Hangzhou, China
**Name and contact information for the trial sponsor {5b}**	**Trial sponsor:** Institute of Cardio-cerebrovascular Disease, Zhejiang Chinese Medical University, Hangzhou, China **Contact name:** Prof. Haitong Wan **Address:** Zhejiang Chinese Medical University, 548 Binwen Road, Hangzhou 310053, China. **E-Mail:** haitongw@163.com
**Role of sponsor {5c}**	The National Natural Science Foundation of China and the National Key R&D Program for Modernization of Traditional Chinese Medicine were not involved in the study design; collection, management, analysis, and interpretation of data; writing of the report; and the decision to submit the report for publication.

## Introduction

### Background and rationale {6a}

In recent years, cardiovascular disease has been one of the major causes of death globally, seriously threatening human life and health [1, 2]. The mortality rate due to cardiovascular diseases accounts for more than 40% of all-cause deaths in China [3]. Coronary artery disease (CAD), a common cardiovascular disease, is the leading cause of morbidity and mortality worldwide [4, 5]. Angina pectoris is the most common symptom of CAD and a major cause of disability. Stable angina pectoris (SAP), as one of the common types of angina pectoris, is a chronic medical condition with an appreciable incidence of acute coronary events and increased mortality [6]. In 2017, the prevalence rate of SAP was 3.6% in China [7], and the numbers are rapidly increasing due to ageing, dramatic lifestyle changes, and expanding populations with multiple risk factors for angina pectoris, such as hypertension and diabetes mellitus. SAP significantly influences patients’ quality of life and places a heavy burden on society and health care systems [8]. As a consequence, it is now urgent that society mounts a comprehensive attack on SAP, harnessing all available resources to slow, arrest, and possibly even reverse the epidemic of cardiovascular diseases [9].

The aim of SAP management is to stop or minimize symptoms and to improve quality of life and long-term morbidity and mortality. Management options include lifestyle advice, drug treatment, and revascularization using percutaneous or surgical techniques [10]. Pharmacological therapy is safe and beneficial for most patients and remains the cornerstone treatment for SAP. Optimizing pharmacological therapy includes nitrates, beta-blockers, calcium channel blockers, antiplatelet agents, angiotensin-converting enzyme inhibitors/angiotensin receptor blockers, statins, etc. [11]. Despite advances in drug treatment and revascularization strategies over several decades, the prognosis of patients with SAP remains poor, and the rates of mortality and rehospitalization are still high [11]. An increasing number of SAP patients are turning to complementary and alternative medicine to address the symptoms and signs of the disease.

The haemodynamic change caused by early coronary artery disease or atherosclerosis is the basic mechanism of SAP [11], which is similar to the perspective of traditional Chinese medicine (TCM) [12]. TCM has been used in China for more than 2000 years and plays a significant role in the treatment of SAP. Using the theoretical basis of TCM and modern complementary and alternative medicine, Buyang Huanwu granules (BYHW) and Naoxintong capsules (NXT) are widely used in the treatment of SAP, and both have been shown to be effective for patients with SAP [13–16]. Modern clinical studies have shown that BYHW and NXT are both similar prescriptions for protecting vascular endothelial function, delaying the process of atherosclerosis, improving haemorheology indices, reducing blood lipids, and exerting anti-inflammatory and antithrombotic effects and have obvious protective effects on myocardial ischaemia-reperfusion injury [14–17]. Although BYHW and NXT have similar efficacy and application, their constituent herbs are different (Table 1), and there are no prospective data comparing the efficacy of the two in the treatment of SAP. Therefore, we designed a randomized, blinded, parallel controlled, multicentre clinical trial to compare the efficacy and safety of NXT and BYHW in the treatment of SAP.

**Table 1:** Characteristics of the investigational product

**Study drug 1: Naoxintong capsule**	
♦ Ingredients: *Astragalus mongholicus* Bunge [Fabaceae; Radix Astragali], *Hirudo nipponia* Whitman [Hirudinidae; Whitmania pigra Whitman], *Boswellia carteri* Birdw., [Burseraceae; Boswellia carterii], *Commiphora myrrha (T.Nees) Engl.,* [Burseraceae; Myrrha], *Salvia miltiorrhiza* Bunge [Lamiaceae; Salviae Miltiorrhizae Radix Et Rhizoma], *Achyranthes bidentata* Blume [Amaranthaceae; Achyranthes], *Neolitsea cassia* (L.) Kosterm. [Lauraceae; Cassia Twig], *Morus alba* L. [Moraceae; Mulberry Twig], *Spatholobus suberectus* Dunn [Fabaceae; Caulis Spatholobi], *Buthus martensii* Karsch [Buthidae; Scorpion];	
♦ Property: capsule; the contents are brown to black brown powder; bitter in taste;	
♦ Specification: 0.4 g/ capsule;	
♦ Bach number: 200593.	
**Placebo 1: Capsule placebo**	
♦ Ingredients: corn starch, silica, caramel (liquid), 2% NXT powder and sunset yellow; ♦ With identical colour, specification, packaging, property of contents and other features with Naoxintong Capsule;	
♦ Bach number: 200501.	
**Study drug 2: Buyang Huanwu granule**	
♦ Ingredients: *Astragalus mongholicus* Bunge [Fabaceae; Radix Astragali], *Angelica sinensis* (Oliv.) Diels [Apiaceae; Angelicae Sinensis Radix], *Paeonia lactiflora* Pall. [Paeoniaceae; Paeoniae Radix Rubra], Lumbricus rubellus (*Oligochaeta*, Lumbricidae), *Ligusticum chuanxiong* Hort. [Apiaceae; Chuan xiong Rhizoma], *Carthamus tinctorius* L. [Asteraceae; Carthami Flos], *Prunus persica* (L.) Batsch [Rosaceae; Semen Persicae].	
♦ Property: granule; the contents are brown to black brown granules; bitter in taste;	
♦ Specification: 5.5 g/ bag;	
♦ Bach number: 200603.	
**Placebo 2: Granule placebo**	
♦ Ingredients: dextrin, 1% Ligusticum chuanxiong, bitterness SA, stevioside, lemon yellow, and chocolate brown; ♦ With identical colour, specification, packaging, property of contents and other features with Buyang Huanwu granule;	
♦ Bach number: 200601.	

### Objectives {7}

The objectives of this study were (i) to evaluate the efficacy of NXT and BYHW in the treatment of SAP and (ii) to test whether NXT is more advantageous than BYHW for treating SAP.

### Trial design {8}

**Fig. 1:**
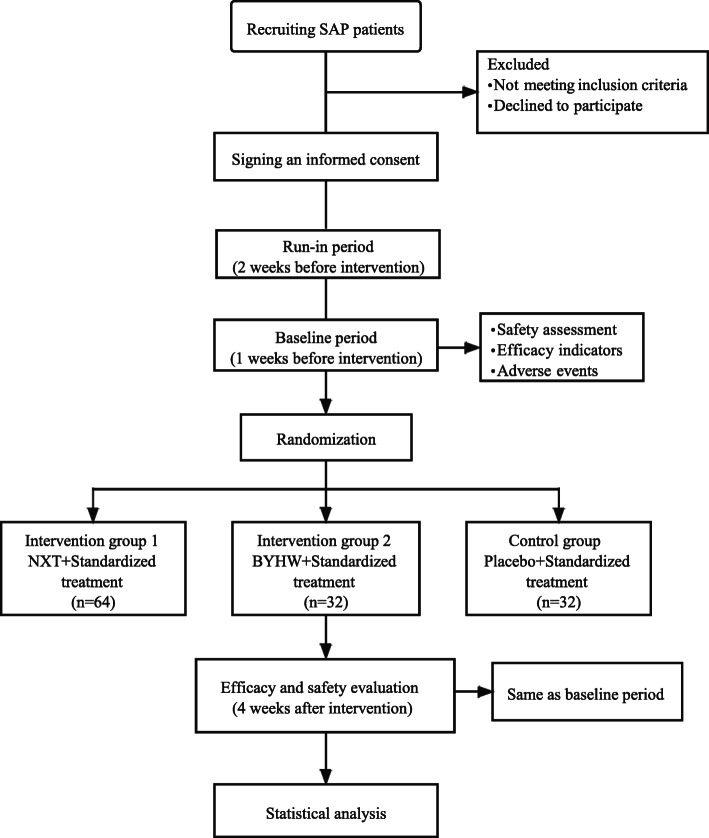
Study flowchart

This study is a randomized, blinded, multicentre parallel-group, placebo-controlled clinical trial. The study consists of a 2-week placebo run-in period, a 1-week baseline period, and a 4-week treatment period. The specific study procedures are shown in Fig. 1.

NXT and BYHW are both prescriptions for Qi invigorating, blood circulation activating, stasis removing, and collaterals dredging [18, 19]. NXT was proposed from BYHW and adds blood-activating herbs and collateral-releasing herbs [18]. Over the past two decades, NXT has been used in treating more than 100 million patients, and published more than 1000 academic papers on its clinical or basic researches were reported [15–18]. From clinical experience, in terms of improving symptoms, NXT is better than BYHW, but there is a lack of clinical research evidence. Therefore, we designed this study to compare the efficacy and safety of NXT and BYHW in the treatment of SAP. In addition, based on ethical considerations, we hope that more patients will benefit from this trial. Thus, the eligible patients will be randomly allocated to intervention group 1 (given NXT and a granule placebo), intervention group 2 (given BYHW and a capsule placebo), and control group (given the granule placebo and the capsule placebo) at a ratio of 2:1:1.

## Methods: participants, interventions, and outcomes

### Study setting {9}

This trial will be conducted at six participating sites, including the Second Affiliated Hospital of Zhejiang Chinese Medical University, Hangzhou Xiaoshan District Hospital of Traditional Chinese Medicine, Lishui Hospital of Traditional Chinese Medicine, Fenyang Hospital of Shanxi Province, Affiliated Hospital of Shanxi Chinese Medical University, and Luohe Hospital of Chinese Medicine.

### Ethical review and general statements

This trial has been approved by the Research Ethics Committee of the Second Affiliated Hospital of Zhejiang Chinese Medical University (No. 2017-Y-070-01) and has been registered at the Chinese Clinical Trials Register (ChiCTR1800015191). To guarantee standardized processes and the safety of all participants, our study will be performed in accordance with the principles of Good Clinical Practices (GCP), recommendations of the CONSORT [20] and SPIRIT [21] statements, and the Helsinki Declaration. All participants will be informed about every detail of the trial and will provide written informed consent according to the Helsinki Declaration before participating. To protect the participants’ privacy, the data forms and eCRFs involved in this study will be maintained in secure storage at the coordinating centre.

### Eligibility criteria {10}

Patients will be included after providing written informed consent and enrolled in the study when the inclusion and exclusion criteria are met.

#### Inclusion criteria

Patients will be eligible for this study if they strictly meet the following criteria.

1. Aged 40 to 75 years, both males and females
2. Diagnosis with SAP by the “Guidelines for diagnosis and treatment of chronic stable angina pectoris in 2007” [22] and the “ACC/AHA2002 guideline update for the management of patients with chronic stable angina” [23]
3. Consistent with the diagnostic criteria of grade I~III SAP
4. History of SAP within 1 month or myocardial infarction (MI) 3 months prior
5. Having undergone coronary revascularization or cardiac pacemaker surgery and, after 3 months of treatment, still having SAP
6. Volunteering to participate and sign informed consent

#### Exclusion criteria

Patients who have any of the following criteria may not participate in this test.

1. History of MI or coronary revascularization or cardiac pacemaker installation within 3 months
2. Abnormal electrocardiogram (ECG) and ST segment decline of more than 1 mm in resting 12-lead ECG
3. Acute MI, severe angina pectoris, pulmonary embolism, aortic dissection, cardiomyopathy, aortic valve stenosis and other heart valve diseases, congenital heart disease, left bundle branch block, or electrolyte disturbance
4. Chest pain caused by severe cardiovascular neurosis, climacteric syndrome, cervical spondylosis, bone and joint diseases, digestive system diseases, and respiratory system diseases
5. Severe liver and kidney dysfunction (including dialysis treatment) and active liver disease (including primary biliary cirrhosis and unexplained persistent liver dysfunction) and/or alanine aminotransferase (ALT), aspartate aminotransferase (AST) > 1.5 times the normal upper limit, creatinine (Cr) > the normal upper limit, and total bilirubin (TBIL) ≥ 2 times the normal upper limit
6. Having other severe cardio-cerebrovascular, liver, kidney, and haematopoietic system diseases or having recently undergone major surgery
7. Psychiatric disorders, severe depression, alcohol addiction, or history of substance abuse
8. Pregnancy or lactation or a positive pregnancy test at the time of admission
9. Allergic constitution or allergy to the study drugs and their ingredients
10. Participation in other clinical trials in the previous 3 months
11. Patients who are judged as inappropriate by investigators

#### Who will take informed consent? {26a}

The recruitment of participants in this study will be carried out in the outpatient clinic. When potential participants are in the clinic, the investigator will evaluate their eligibility according to the inclusion and exclusion criteria. Then, trained study staff (physicians or psychologists) will provide verbal information about the trial, answer questions, and obtain written informed consent from potential trial participants. After informed consent obtained, the participants will enter the run-in period.

#### Additional consent provisions for collection and use of participant data and biological specimens {26b}

On the consent form, participants will be informed that their data will be anonymized. This trial does not involve collecting biological specimens for storage.

### Interventions

#### Explanation for the choice of comparators {6b}

Given the complexity of SAP and the urgent need for evidence-based therapy approaches, we chose to add TCM on the basis of standardized treatments and compare the efficacy of NXT and BYHW in relieving stable angina symptoms (including the frequency of angina attacks, duration and degree of pain).

The two intervention groups, i.e. the NXT group and BYHW group, will be compared to the placebo group to evaluate the efficacy of NXT and BYHW in the treatment of SAP. Furthermore, the NXT group will be compared to the BYHW group to evaluate whether NXT is more advantageous than BYHW for treating SAP.

#### Intervention description {11a}

Eligible participants will experience a 2-week run-in period, a 1-week baseline period, and a 4-week treatment period. The interventions during the treatment period are shown in Table 2, and details are as follows:

**Table 2:** Intervention implementation plan

Intervention groups	Naoxintong capsule	Capsule placebo	Buyang Huanwu granule	Granule placebo
**Intervention group 1**	✓			✓
**Intervention group 2**		✓	✓	
**Control group**		✓		✓

Intervention group 1 will receive NXT (0.4 g/capsule, op, 4 capsules/tid) and a granule placebo (5.5 g/bag, op, 1 bag/tid).

Intervention group 2 will receive BYHW (5.5 g/bag, op, 1 bag/tid) and a capsule placebo (0.4 g/capsule, op, 4 capsules/tid).

The control group will receive the capsule placebo (0.4 g/capsule, op, 4 capsules/tid) and the granule placebo (5.5 g/bag, op, 1 bag/tid).

Placebo drugs will be given for 2 weeks during the run-in period, followed by the appropriate medication for a 4-week intervention period.

Standardized treatment: During the run-in and intervention periods, according to the guidelines for SAP treatment, participants will receive standardized treatment consisting of aspirin, statins, ACEIs, and/or ARBs. Substitution with TCM having a similar composition and/or efficacy to NXT or BYHW is not permitted. If instances of an angina attack occur, participants should take one nitroglycerine tablet under the tongue at a time and can repeat one tablet every 5 min until the pain is relieved. If the pain persists after the total amount reaches three tablets within 15 min, medical attention will be immediately requested.

The following concomitant therapies will be permitted, provided the regimen has been stable for the specified period of time prior to the first dose of study drug and the dose remains the same with no increase or decrease during the study treatment period:

1. Beta blockers, antiplatelet therapy, statins, angiotensin-converting enzyme inhibitors, and angiotensin receptor blockers will be allowed provided the dose has been stable for at least 4 weeks prior to the run-in period. If instances of an angina attack occur, nitroglycerine tablets can be used sublingually.
2. During the intervention period, when combined medication is required for other diseases, the investigator should record the name of the medication, the reason for the medication, and the time of medication in detail.
3. If infection, hyperglycaemia, hypertension, etc., are present in the intervention period, symptomatic drugs will be allowed provided the therapy does not affect the efficacy and safety of the test drug. All drugs used must be recorded for analysis and reporting in the summary.

The following medications are prohibited throughout the duration of the study treatment period. If banned drugs are used, those subjects will be withdrawn from the study.

1. Long-acting nitrates
2. Chinese medicinal preparations that are used for the treatment of coronary heart disease or may have the effects of promoting Qi, promoting blood circulation and removing blood stasis
3. Anticoagulant drugs
4. Other drugs that may affect the evaluation of test drugs

In this study, NXT, BYHW, the capsule placebo, and the granule placebo are provided by Shaanxi Buchang Pharmaceutical Co. Ltd. The capsule and granule placebos are similar to the study drug in smell, colour, specification, packaging, property of contents, and other features.

#### Criteria for discontinuing or modifying allocated interventions {11b}

Participants are allowed to withdraw at any time without having to give a reason. The withdrawal criteria are as follows: (1) participants request withdrawal from the study; (2) there is exacerbation or deterioration that is clearly related to the treatment; (3) an allergic reaction that is clearly associated with the study drug occurs; (4) participants show comorbidities, complications, adverse events (AEs), or serious adverse events (SAEs) during the trial; (5) there is use of forbidden drugs or receipt of prohibited treatment; (6) participants show poor adherence, or the amount of drug used does not meet the regulations (< 80%); and (7) blinding is uncovered or emergency unblinding is required.

If a subject withdraws from the trial, no additional data will be collected, but the existing data will be used for statistical analysis. The time of treatment or trial discontinuation and the reason for withdrawal will be documented on the CRF. Drop-out patients will not be replaced.

#### Strategies to improve adherence to interventions {11c}

Low adherence can have a substantial effect on statistical power and biased estimates of an intervention’s effect. To improve adherence to intervention protocols, during the treatment phase, we will distribute medicines once a week to evaluate the participant’s drug consumption and monitor adherence in a timely manner. Face-to-face adherence-reminder sessions will take place during the medicine dispensing process. After treatment, the drug package will be returned to the researchers. Compliance will be self-reported by participants and will be assessed by investigators at each visit. Compliance is calculated using the following formula: compliance = (actual amount of internal use/total amount of internal use) × 100%. Poor compliance is predefined as compliance < 80%. After 28 days of treatment, participants with poor compliance will be removed from the per protocol set (PPS).

#### Relevant concomitant care permitted or prohibited during the trial {11d}

Concerning standardized treatment, during the run-in period and treatment period, aspirin, statin lipid-lowering drugs, angiotensin converting enzyme inhibitors (ACEIs), or angiotensin receptor blockers (ARBs) should be used. TCM formulations with a similar composition and efficacy to those of study drugs will not be allowed to be used. Researchers should record the concomitant medication accurately and maintain dose stability during the trial. The participants should be clinically stable for the last 1 month or receiving standardized treatment for ≥1 month with no modification of dosage or intravenous administration.

Concerning emergency care, in the event of an acute angina pectoris attack during treatment, participants should be treated with nitroglycerine first (one tablet at a time, by sublingual administration, which can be repeated every 5 min until the pain is relieved). If the pain persists after the total amount reaches 3 tablets in 15 min, medical attention should be sought immediately. The treatment status should be recorded. If the participant’s condition deteriorates during treatment and it is not advisable to continue the trial, the researcher should consider terminating the trial and switching to surgery or another type of clinical treatment, and the patients will be classified in the analysis as “treatment ineffective”.

#### Provisions for post-trial care {30}

If SAEs occur during the study period, the participants are also required to be followed up after the study period. Appropriate measures will be taken to fully protect the interests of participants, such as outpatient or inpatient care or referrals to other specialists.

### Outcomes {12}

#### Primary outcome

The primary outcome is the efficacy rate of stable angina symptom score classification [24]. The stable angina symptom score includes the attack frequency, duration, activity level of effort-induced angina pectoris, and pain degree. The indicators of the baseline period (0 weeks) and treatment period (28 days) will be compared.

#### Secondary outcomes

The secondary outcomes are the effect on ECG [25], change in Seattle Angina Questionnaire (SAQ) scores [26], and nitroglycerine consumption during the treatment period. Patients will be asked to complete an SAQ (at the baseline period and at the end of the 4-week treatment), which is a 19-item self-administered questionnaire, to measure five dimensions of health. Researchers will record nitroglycerine consumption during the treatment period and compare this among groups.

#### Safety outcomes

Safety evaluations will include vital signs (heart rate, respiration, body temperature, blood pressure); clinical laboratory tests (routine blood test, routine urinalysis, serum biochemistry, blood coagulation index, fasting blood glucose), AEs, and SAEs.

The five elements of the primary outcome, secondary outcomes, and safety outcomes are shown in Table 3.

**Table 3:** The five elements of the outcomes

Domain	Specific measurement	Specific metric	Method of aggregation	Time-points
***Primary outcome***				
Stable angina symptom score	Symptom score standard classification [22]	Difference between two groups	Proportion	Baseline, 28 days after treatment
***Secondary outcomes***				
ECG parameters	Classification of 12-lead ECG improvement [23]	Difference between two groups	Proportion	Baseline, 28 days after treatment
SAQ scores	SAQ [24]	Difference in the change between groups	Mean/ median	Baseline, 28 days after treatment
Nitroglycerin consumption	Count	Compare the difference between groups	Median	Baseline, 28 days after treatment
***Safety outcomes***				
Vital signs	Heart rate, respiration, body temperature, blood pressure	Compare the difference between groups	Mean/ median	Run-in period, baseline, 28 days after treatment
Clinical laboratory tests	Routine blood test, routine urinalysis, serum biochemistry, blood coagulation index, and fasting blood glucose	Compare the difference between groups	Mean/ median	Baseline, 28 days after treatment
AEs, SAEs	Common Terminology Criteria for AEs version 4.03.	Compare the difference between groups	Proportion	Baseline, 28 days after treatment

*ECG* electrocardiogram, *SAQ* Seattle Angina Questionnaire

### Participant timeline {13}

The measurement items and timeline for data collection in this study are displayed in Table 4.

**Table 4:** Measurement items and time points for data collection

Item	Run-in period (−14 ± 1) day	Baseline period (−7~0) days	Intervention period (28 ± 4) days
**Basic information**			
Informed consent	×		
Inclusion/exclusion criteria		×	
Demographic data	×		
Randomization		×	
Record medical history and allergy history	×		
Record complication and symptom	×		
Record concomitant medication	×		
Access to the “Doctor Tao” platform	×		
Urine pregnancy test	×		
**Safety assessment**			
Vital signs and physical examination	×	×	×
Blood and urine routine examination		×	×
Liver function (ALT, AST, AP, TBIL, γ-GT)		×	×
Kidney function (SCr, BUN)		×	×
Coagulation function (PT, APTT, TT, FIB, INR)		×	×
Fasting blood glucose		×	×
**Efficacy indicators**			
Stable angina symptom score		×	×
Electrocardiogram		×	×
Seattle Angina Questionnaire		×	×
Nitroglycerin consumption	×	×	×
**Other works**			
Drug distribution	×	×	
Drug recycling		×	×
Record adverse events and combined medication		×	×
Compliance judgement		×	×

*ALT* alanine aminotransferase, *AST* aspartate aminotransferase, *AP* alkaline phosphatase, *TBIL* total bilirubin, *γ-GT* γ-glutamyl transpeptidase, *SCr* serumcreatinine, *BUN* blood urea nitrogen, *PT* prothrombin time, *APTT* activated partial thrombolastin time, *TT* thrombin time, *FIB* fibrinogen, *INR* international normalized ratio

### Sample size {14}

The sample size is calculated using PASS 15 software (NCSS, Kaysville, UT) and driven by the efficacy rate of stable angina symptom score improvement. According to the referenced literature [27, 28], the efficacy rate in relieving stable angina symptoms of NXT is expected to be 85%, 70% for BYHW, and 50% for placebo.

The PASS parameters are as follows: “Proportions>Contingency Table (Chi-Square Tests)>Chi-Square Tests”, “Chi-Square Effect Size Estimator> Contingency Table”. Input the percentage values: placebo = 50%, BYHW = 70%, NXT = “85%”, copy effect size = “0.308210”. Design solve for “sample size”, DF = 2, Power = 0.8, Alpha = 0.05, input effect size = “0.308210”. Then we can get numeric results: *N* = 102, Chi-square = 9.6893, Alpha = 0.05000, Beta = 0.19763. Considering a drop rate of 20%, the dropout-inflated sample size = 128, and expected number of dropouts = 26. According to the ratio of 2:1:1, there will be 64 cases in intervention group 1, 32 cases each in the intervention group 2 and control group. Since this study intends to further compare the NXT and BYHW groups, according to NXT = 85%, BYHW = 70%, W = 0.3082, DF = 1, and the sample size of NXT and BYHW calculated in the previous step, we can reach a power of 0.85540.

### Recruitment {15}

Potential participants will be recruited from outpatients and inpatients in the cardiovascular department by advertisement, verbal promotion by researchers, hospital posters, and doctor referrals.

## Assignment of interventions: allocation

### Sequence generation {16a}

Eligible participants will be randomized in a 2:1:1 ratio into intervention group 1, intervention group 2, or the control group based on a computer-generated (SAS 9.4 statistical software package PROC PLAN process) random sequence. The randomization sequence will be recorded by staff at the sponsor unit, and the randomized code of the trial drug is the unique identification code of participants. Eligible participants will be randomized in blocks of eight within each site and stratified by site.

### Concealment mechanism {16b}

The allocation sequence will be implemented through sequentially numbered, opaque, sealed envelopes that are concealed until the interventions are assigned to a participant. Participants withdrawn from the trial will retain their randomization number (identification codes) if already given. New subjects must always be allotted a new randomization number.

### Implementation {16c}

During this trial, the statistician will generate the allocation sequence, and the investigators will enrol participants and assign participants to interventions based on information obtained upon opening the randomization number envelope.

## Assignment of interventions: blinding

### Who will be blinded {17a}

To minimize ascertainment bias, all researchers, participants, physicians, drug administrators, dispensing nurses, and statisticians will be blinded to the type of treatment until the study is completed.

### Procedure for unblinding if needed {17b}

Unblinding will be available if participants experience severe adverse events (SAEs) or need to be rescued in an emergency situation. Once unblinded, the participant will withdraw from the study. Researchers should report the reasons to the inspector within 24 h. The precise cause of unblinding, date of adverse events (AEs), treatment situation, and results must be recorded in electronic case record forms (eCRFs).

## Data collection and management

### Plans for assessment and collection of outcomes {18a}

According to the requirements of GCP, the investigator shall fill in data to CRFs accurately, completely, and in a timely manner based on original observations of the subjects. The auditor shall monitor whether all CRFs have been completed correctly and are consistent with the source data and shall pose questions at any time in case of any problem. If errors and omissions are made, the researcher shall be corrected promptly. All data will be stored securely in line with local data management arrangements. Data management will be implemented by Beijing Yilian Zhongkang Technology Co., Ltd.

### Plans to promote participant retention and complete follow-up {18b}

To promote participant retention, at each medicine distribution visit, participants will be scheduled by phone, sent messages, and called the day before. Missed visits will be rescheduled and followed up. The sponsor will provide compensation for transportation costs.

### Data management {19}

CRFs are paper-based. Source data will be collected at each site from the investigator and will be transferred into the CRFs. Source data will be stored in the respective centre. The destruction of any paper files will occur at least 5 years from the termination of the study and will be authorized by the sponsor and the principal investigator.

EpiData Software version 3.1 (the EpiData Association, Odense, Denmark) will be used to store outcome measurement data. Two personnel will undertake double-entry and proofreading independently to ensure the accuracy of the data input from the CRF to EPidata. Electronic data files will be backed up and password protected.

### Confidentiality {27}

Participant identification numbers are used to track data collection documents. Interventionists’ paper documents (CRFs) are stored in a locked file cabinet. The CRFs only use the first letter of the name to identify the participant and do not include the participant’s home address. Access to electronic data is password protected and restricted to the research team. Data exchanged between research sites are deidentified, encrypted, and password protected.

This protocol, CRFs, and other documents and materials related to the trial will be kept strictly confidential and will not be disclosed to third parties unless expressly agreed upon by the principal investigator in advance. Staff of the investigators involved in this trial are also bound by the agreement.

### Plans for collection, laboratory evaluation, and storage of biological specimens for genetic or molecular analysis in this trial/future use {33}

Participants are asked to give explicit consent for blood sample collection and use. For blood sample collection, subjects will be required to fast for 10 h before each collection; 5 mL of blood will be drawn and centrifuged, and the serum will be stored in an Eppendorf tube at − 70 °C.

## Statistical methods

### Statistical methods for primary and secondary outcomes {20a}

The statistical analysis plan will be specified before data analysis. Statistical Analysis System v9.4 (or a higher version) will be used for statistical analysis. Professional statisticians who are independent of all other processes in our study will carry out the analyses.

For continuous variables, we will calculate the mean, standard deviation, median, minimum, maximum, and interquartile range. For categorical variables, we will report various frequencies or percentages. The chi-square test or Fisher’s exact test will be used for categorical variables. For variables with a normal distribution, intergroup comparisons will be analysed with one-way ANOVA, whereas pairwise comparisons before and after treatment will be performed with Student’s *t* test. For data that do not have a normal distribution, intragroup or intergroup differences before and after treatment will be analysed by the Wilcoxon rank-sum test. The proportion of patients with AEs will be compared using the chi-square test or Fisher’s exact test.

### Interim analyses {21b}

Since the number of recruited cases is not large, no interim analysis is planned.

### Methods for additional analyses (e.g. subgroup analyses) {20b}

We will include sex, centre, complications, and concomitant medication as covariates in an analysis of covariance (ANOVA) for significant differences between groups by reducing the error variance.

### Methods in analysis to handle protocol non-adherence and any statistical methods to handle missing data {20c}

This study will be consistent with the CONSORT statement and intention-to-treat (ITT) principle, and the last observation carried forward method will be used for missing values. The full analysis set (FAS) is defined as all randomized patients who receive at least one dose of study medication. The per-protocol analysis set is defined as all eligible patients who are at least 80% compliant with the study drug and do not take the prohibited drugs during their time in the study. Analysis of the primary outcome and curative effect will be carried out using full-analysis-set and per-protocol-set approaches. The safety analysis set will include all randomized patients who have accomplished at least one study visit. Participating centres will be required to sum up the participant numbers in each centre and list participants who have been removed from the per-protocol set.

### Plans to give access to the full protocol, participant-level data {31c}

After the publication of the results of this trial, the deidentified version of the database will be available upon reasonable request from the principal investigator.

## Oversight and monitoring

### Composition of the coordinating centre and trial steering committee {5d}

The trial steering committee is composed of the principal investigator (Wan), on-site principal investigators of each hospital, and coinvestigators. Questions that arise during the research process will be submitted to the committee for decision-making. Principal investigators and on-site principal investigators are responsible for the overall management and will coordinate operations of their respective centres. The principal investigator (Wan) is responsible for overall management of the financial and administrative operations. The on-site principal investigators are responsible for the research coordination and implementation of their respective sites.

### Composition of the data monitoring committee, its role, and reporting structure {21a}

This study commissioned Guoxin Pharmaceutical Technology (Beijing) Co., Ltd., to carry out clinical supervision and data monitoring and set up safety officers to be responsible for security review. All the medical records of the subjects will be carefully assessed, and all harms and complications of the treatment will be reported. The harms will be categorized into AEs and SAEs as described in the AEs section. We will not conduct auditing between the participant sites during the trial. Safety officers will review reports on recruitment, retention, and safety information for all participants twice per year, including AEs and SAEs.

### Adverse event reporting and harms {22}

Both NXT and BYHW are Chinese medicines for promoting blood circulation and removing blood stasis. Their side effects may include mild gastrointestinal reactions such as stomach pain, nausea, and loss of appetite in a small number of patients. Very few patients may have symptoms such as skin itching, peeling, papules, drowsiness, upset, or headache, which can disappear after stopping the drug. As long as the medication is used correctly, the side effects will be appropriately reduced.

The investigator should explain and ask the participants to truthfully reflect changes in their condition after the medication, avoiding leading questions. While observing the curative effect, attention should be given to observing adverse reactions, AEs, or unexpected side effects (including symptoms, signs, laboratory tests). Regardless of whether the adverse reaction or AE is related to the trial drug, it should be recorded in detail, including the time of occurrence, symptoms, signs, severity, duration, laboratory test indicators, treatment methods and results, elapsed time, follow-up time, etc., and the combined medications should be described in detail for analysis of the correlation between the AEs and the trial drug.

During the treatment period, all AEs and SAEs should be monitored and recorded at every study visit. Once AEs occur, the researcher will evaluate whether they are related to the test drug and will judge the severity of the AEs. In cases of SAEs, the participating patients are asked to take immediate measures to protect their safety and report the events to the ethics committee within 24 h. The classification and coding of AEs are formulated according to the Common Terminology Criteria for AEs version 4.03.

### Frequency and plans for auditing trial conduct {23}

The trial includes close monitoring by the principal investigator, on-site principal investigators, and external agency. Annual progress reports are provided to the sponsor and principal investigator.

### Plans for communicating important protocol amendments to relevant parties (e.g. trial participants, ethical committees) {25}

Any changes to the eligibility criteria, outcomes, or analyses will be reviewed by the trial steering committee and updated in ClinicalTrials.gov.

### Dissemination plans {31a}

The results of this study will be disseminated through peer-reviewed publications; presentations at local, national, and international academic conferences; and reports to funders. In addition, a summary of the primary outcome findings will be created in English and Chinese and shared with the study participants.

## Discussion

SAP is closely related to the risk of cardiovascular death and recurrent MI and h as an important influence on physical function and quality of life [29]. In recent years, the goal of treatment has been to improve symptoms and quality of life, prevent cardiovascular events, and reduce the mortality and hospitalization rate of SAP. Nitrates, beta-blockers, calcium channel blockers, antiplatelet agents, angiotensin-converting enzyme inhibitors/angiotensin receptor blockers, statins, etc., have been shown to be beneficial [11]. Despite some progress in the field of SAP treatment, the current prevalence and mortality remain high. There is still a need to generate new treatments and develop new drugs to achieve breakthroughs in SAP treatment.

TCM has gained increasing acceptance worldwide in recent years [27], and its practitioners have accumulated rich experience in the long-term medical practice of preventing and treating SAP; moreover, the understanding of its use for SAP is also deepening. There have been some studies and reports on the treatment of heart failure with TCM. NXT and BYHW are both drugs for the treatment of SAP with insufficient heart-Qi and collaterals obstruction. BYHW was proposed by Wang Qingren in the Qing dynasty and has been used in the treatment of cardiovascular and cerebrovascular diseases for nearly 100 years. Under the guidance of the theory of “Inheriting the Essence, and Adherence and Innovation”, due to a weak ability of BYHW to activate blood circulation and dredge collaterals, NXT was proposed and manufactured by Buchang Pharmaceutical Co., Ltd. NXT (national medicine permission number: Z20025001), obtained drug production approval in 1993, was awarded National Chinese Medicine Protection Certificate in 2014, and was included in the National Basic Drug List (2012 edition) and the Chinese Pharmacopoeia (2015 edition). Over the past two decades, NXT has been used to treat more than 100 million patients and can significantly improve the total efficacy rate against cardiovascular/cerebrovascular diseases [21].

Modern clinical studies have shown that BYHW can significantly improve the effectiveness of angina pectoris; reduce the attack duration, degree of pain, and nitroglycerine consumption; and improve the blood lipid index [28]. NXT is a next-generation innovation of the basic formula of BYHW. Li et al. systematically evaluated the efficacy and safety of NXT in the treatment of SAP, which included 44 studies and 5130 patients. The results showed that NXT can significantly improve ECG efficacy, relieve angina symptoms, and improve clinical efficacy [30]. In addition, there is evidence that both NXT and BYHW can protect vascular endothelial function, delay the process of atherosclerosis, improve haemorheological indicators, reduce blood lipids, have anti-inflammatory and antithrombotic effects, and exert obvious protective effects against myocardial ischaemia-reperfusion injury [14–17]. Since the two Chinese patent medicines have similar indications, mechanisms, and clinical efficacy for SAP, we designed a protocol to observe the efficacy and safety of NXT and BYHW in the treatment of SAP.

However, this study has limitations. First, AEs will only be recorded and processed during the 4-week intervention period, which is relatively short, but these short-term results could encourage further prospective studies with different treatment regimens and longer follow-up periods. Second, due to the short intervention period, this study lacks long-term observations of mortality and cardiovascular events. A large-scale and long-term future study that assesses mortality and cardiovascular events as the primary endpoint is expected.

In conclusion, under rigorous design and strict quality control, we expect that the results of this study will provide valuable evidence to determine whether the effect of NXT is superior to that of BYHW for improving the symptoms of patients with SAP.

## Trial status

The trial was initiated in June 2018 and will be completed by June 2022. The recruitment is in progress. No analysis has been carried out since the beginning of the trial.

## Data Availability

Data and materials generated during the current study are available from the corresponding author upon request.

## Abbreviations

ACEI: Angiotensin converting enzyme inhibitors
AEs: Adverse events
ALT: Alanine aminotransferase
AP: Alkaline phosphatase
APTT: Activated partial thrombolastin time
ARB: Angiotensin receptor blockers
AST: Aspartate aminotransferase
BUN: Blood urea nitrogen
BYHW: Buyang Huanwu granules
CAD: Coronary artery disease
Cr: Creatinin
ECG: Electrocardiogram
eCRFs: Electronic case record forms
FIB: Fibrinogen
GCP: Good Clinical Practices
INR: International normalized ratio
ITT: Intention-to-treat
MI: Myocardial infarction
NXT: Naoxintong capsule
PPS: Per protocol set
PT: Prothrombin time
SAEs: Severe adverse events
SAP: Stable angina pectoris
SAQ: Seattle Angina Questionnaire
SCr: Serum creatinine
TBIL: Total bilirubin
TCM: Traditional Chinese medicine
TT: Thrombin time
γ-GT: γ-glutamyl transpeptidase
